# Stereotactic Body Radiotherapy as a Salvage Therapy after Incomplete Radiofrequency Ablation for Hepatocellular Carcinoma: A Retrospective Cohort Study

**DOI:** 10.1155/2020/4835653

**Published:** 2020-05-28

**Authors:** Yizhen Fu, Mian Xi, Yangxun Pan, Jinbin Chen, Juncheng Wang, Shiliang Liu, Li Xu, Zhongguo Zhou, Mengzhong Liu, Minshan Chen, Lei Zhao, Yaojun Zhang

**Affiliations:** ^1^Sun Yat-sen University Cancer Center, State Key Laboratory of Oncology in South China, Collaborative Innovation Center for Cancer Medicine, Guangzhou, Guangdong 510060, China; ^2^Department of Liver Surgery, Sun Yat-sen University Cancer Center, Guangzhou, Guangdong 510060, China; ^3^Department of Radiation Oncology, Sun Yat-sen University Cancer Center, Guangzhou, Guangdong 510060, China

## Abstract

Residual tumor tissue after radiofrequency ablation (RFA) is inevitable in clinical practice, and the optimal management of residual tumor after RFA has not been established. To evaluate the efficiency and toxicity of stereotactic body radiotherapy (SBRT) as a salvage therapy after incomplete RFA for hepatocellular carcinoma (HCC), we retrospectively included 32 HCC patients with an initial incomplete response (iIR) to RFA from May 2011 to August 2018. An iIR was defined as the presence of residual enhancement on CT or MRI one month after RFA treatment. The primary endpoint was local tumor control (LTC); the secondary endpoints included progression-free survival (PFS), overall survival (OS), and toxicity. All patients fulfilled 6 fractions of SBRT as planned, with dosages ranging from 30 Gy to 54 Gy. The objective response rate (ORR) was 50.0%. The 1- and 2-year LTC rates were 86.6% (95% CI, 74.3% to 98.9%) and 74.7% (95% CI, 55.9% to 93.5%), respectively. Fewer times of prior treatments was associated with better LTC (HR = 11.7, *P*=0.026). The 1- and 2-year PFS rate were 69.9% (95% CI, 53.4% to 86.4%) and 52.7% (95% CI, 33.1% to 72.3%), respectively. A higher Child-Pugh score was the only independent risk factor for tumor progression (HR = 5.17, *P*=0.012). The 1- and 3-year OS rate were 85.6% and 67.1%, respectively. Only two patients suffered grade 3 adverse events, and none experienced grade 4 or 5 events. In conclusion, for HCC patients confirmed to have an iIR to prior RFA, with compensated liver function, SBRT provided favorable LTC and OS along with acceptable toxicity.

## 1. Introduction

Liver cancer is the sixth most commonly diagnosed cancer and the fourth leading cause of cancer death worldwide in 2018, with approximately 841,000 new cases and 782,000 deaths annually. Hepatocellular carcinoma (HCC) is the most common primary liver cancer, accounting for 75%–85% of cases [1]. For early-stage HCC, transplantation, surgical resection, and ablation are considered as curative treatments [2].

Radiofrequency ablation (RFA) provides comparable results to liver resection with fewer complications in early-stage HCC. Technical success, also known as an initial complete response (iCR), which refers to the thorough coagulation necrosis of the treated lesion, is reported to be associated with tumor control and patient survival [3, 4]. However, the iCR rate fails to reach 100% even in case of small HCC lesions, since some HCC lesions are located beneath the liver capsule or adjacent to vessels (leading to the so-called “heat-sink effect”) [5]. Residual tumor tissue after RFA treatment, or an initial incomplete response (iIR), is inevitable in clinical practice even with skilfully applied ablation techniques and advanced approaches. Several studies have reported an initial failure rate of RFA ranging from 5.2% to 16.9% [3, 6–8]. Most of these patients underwent repeated RFA after the confirmation of an iIR [3–9], yet the underlying causes of RFA failure have not been settled, and the iIR rate of repeated RFA is still approximately 7% (6.6 to 7.8%) [7, 8]. Given that an iCR is a major predictor of survival, treatment of the remaining lesion is as essential as treatment of the primary [3, 4]. Little research has focused on treatment after prior RFA failure, and the optimal management for residual tumor tissue owing to an iIR to RFA has not been established.

Stereotactic body radiation therapy (SBRT) is a treatment modality that involves the delivery of very high individual doses of radiation to tumors with high precision within a single or a small number of fractions [10]. Due to its high geometric precision and accuracy and the consequently lower radiation exposure of nontargeted tissue, along with fair local control and feasible toxicities [11–13], SBRT has emerged as an alternative treatment to conventional therapies. Retrospective studies have shown equivalent overall survival (OS) and superior local control with SBRT compared to RFA [14, 15]. Since SBRT has been proven to be efficient in small HCC for curative intension, we rationally assume that SBRT should provide fair local control and favor OS if it is applied as a salvage operation to residual lesions after RFA. As far as we know, the outcomes of SBRT in treating residual lesions remain unknown. Thus, in this observational study, we reviewed patients who underwent SBRT after confirmation of an iIR to prior RFA treatment, evaluated the efficiency and toxicity of SBRT, and assessed factors potentially influencing tumor control and patient survival.

## 2. Materials and Methods

### 2.1. Data Source

This study was approved by the ethics committee of the Sun Yat-sen University Cancer Center (SYSUCC). Informed consent was impossible to obtain because this was a retrospective study. However, written informed consent for the use of data for research purposes was signed before each treatment. We reviewed the medical records of HCC patients who suffered initial incomplete necrosis after RFA and consequently underwent SBRT between May 2011 and August 2018 at the SYSUCC. The patients' baseline information, including age, sex, BMI, and preceding liver-directed treatments, was obtained from clinical records. Imaging data and laboratory investigation results were collected from the database of the SYSUCC.

### 2.2. Study Population

The object of this study was HCC patients. The diagnosis of HCC was confirmed by biopsy or imaging analysis showing intense contrast uptake during the arterial phase followed by contrast washout in the venous or delayed phase on dynamic computed tomography (CT) or magnetic resonance imaging (MRI).

According to the RFA protocol of the SYSUCC, an early follow-up examination was conducted one month after the RFA operation; thus, an iCR, or technical success of RFA, was defined when the ablated tumor was completely replaced by a necrosis zone with no enhancing tissue at the tumor site, while an iIR, or failed RFA, was defined by the presence of residual enhancement on CT or MRI at the early follow-up time [7, 9]. Once an iIR to previous RFA was confirmed, further treatment was proposed by a multidisciplinary team. Tumor characteristics, liver function, patient performance, and comorbidities were considered to develop the best treatment plan. Generally, the multidisciplinary team proposed SBRT to patients with an iIR who experienced repeated tumor recurrence or whose tumor abutted intrahepatic vessels or the liver capsule. Those who were verified as having an iIR to RFA and subsequently underwent SBRT treatment were enrolled in this retrospective study.

The inclusion criteria were as follows: ≥18 years old; Child-Pugh (CP) A liver function; and Karnofsky performance score ≥ 60 before SBRT implementation. The exclusion criteria were as follows: bilirubin ≥3 times the upper limit of normal; AST or ALT ≥6 times the upper limit of normal; serum creatinine greater than 200 *μ*mol/L; international normalized ratio ≥1.3; hemoglobin less than 90 g/L; platelets less than 80,000/*μ*L; clinical ascites; and previous irradiation to the right upper abdomen. Extrahepatic metastasis was permitted.

To investigate whether the tumor location influenced the efficacy of SBRT, we classified the residual HCC nodules based on their intrahepatic locations shown on CT or MRI before the initiation of SBRT. According to their relation to intrahepatic vessels, the target lesions were classified as perivessel HCC lesions and non-perivessel HCC lesions. Perivessel HCC was defined as an index tumor with any contact with first- or second-degree branches of an intrahepatic vessel that was 3 mm or greater in axial diameter. Subcapsular HCC was defined as a target tumor adjoining the liver capsule on axial or coronal sections with a distance from the hepatic capsule to the tumor margin less than 1 mm. Otherwise, the lesion was considered non-subcapsular [16].

### 2.3. SBRT Treatment

Patients underwent CT simulation with vacuum pillows used to individually immobilize the torso during radiotherapy. Four-dimensional CT combined with a respiratory gating system was used to enable accurate motion management. The gross target volume (GTV) was exactly coincident with the tumor images or enhancing vessel thromboses on CT/MRI. The clinical target volume (CTV) included a 5 mm expansion around the GTV. Statistics obtained during free breathing, deep inspiration, and deep expiration were applied to generate an internal target volume (ITV) that accounted for respiratory motion. Finally, to account for mechanical error and unpredictable changes during each fraction, another margin was added to the ITV to form a patient-specific planning target volume (PTV). The maximal allowable dose to 0.5 mL of the esophagus, duodenum, stomach, and bowel was 30 Gy, that to the spinal cord was 27 Gy, and that to the heart was 52.5 Gy. The chest wall received no more than 35 Gy per 30 mL [13, 14, 17, 18]. Dosimetry was prescribed to the isodose surface covering 99.5% of the PTV, and regional underdosing was allowed to meet normal tissue limits. Patients received 30–54 Gy radiation in 6 fractions every other day with a 6 to 8 MV X-ray beam applied using the Elekta Precise Treatment System (Elekta AB, Stockholm, Sweden) or the Elekta Versa HD System (Elekta AB, Stockholm, Sweden). Cone-beam CT was performed before each treatment for the patients, and image-guided radiation therapy was used for repositioning.

### 2.4. Evaluation

Patients underwent triphasic liver MRI or CT scan one month after finishing all SBRT fractions and were followed up every 3 months in the first year and every 6 months thereafter. Along with imaging data, clinical symptoms and signs were evaluated. Blood specimens were collected at each follow-up time to test for blood system disorders, hepatobiliary system disorders, and tumor markers. Patients who showed suspicious clinical or tomography features of extrahepatic metastasis underwent appropriate further imaging examinations for confirmation. When recurrence after SBRT was detected during the follow-up time, the patient received further local or systemic therapy.

The tumor response after SBRT was assessed according to modified Response Evaluation and Criteria in Solid Tumors (mRECIST) [2, 19]. Local tumor control (LTC) was defined as the absence of progressive disease within or at the PTV margin, while the presence of progressive disease was considered local tumor progression (LTP). New hepatic lesions that emerged outside the PTV margin were classified as intrahepatic distant recurrence (IDR) [11, 13, 14, 17, 20]. The primary endpoint of this study was LTC, and the secondary endpoints included progression-free survival (PFS), overall survival (OS), and toxicity. PFS was defined as the time from the start of SBRT to the earliest event (i.e., LTP, IDR, or extrahepatic recurrence (ER)) pinpointed on radiology. Adverse events were assessed in the first follow-up procedure by the Common Terminology Criteria for Adverse Events (CTCAE), version 5.0. Dose-limiting toxicity (DLT) was considered any grade 4 or 5 liver, intestinal, or hematopoietic system toxic effects or radiation-induced liver disease (RILD), which consisted of hepatomegaly, anicteric ascites, and elevated alkaline phosphatase [13, 17, 21].

### 2.5. Statistical Methods

All recruited patients were enrolled in the calculation of LTC, PFS, and OS. The LTC, PFS, and OS curves were summarized by the Kaplan-Meier method. Raw data for continuous variables, consisting of age, BMI, number of prior treatments, and alpha-fetoprotein (AFP) level and dose to the PTV, were recorded and further converted into categorical data by dichotomizing them by the median values (AFP level: 200 ng/mL). Survival curves of different groups were compared by the log rank test for univariate analysis. The effects of covariates on LTC, PFS, and OS were evaluated by the hazard ratio (HR) using Cox proportional hazards regression models. Variables with *P* values less than 0.1 and those that may have an impact on tumor progression or survival based on clinical experience were included in the multivariate analysis. Continuous variables were compared using the Mann-Whitney test, and categorical variables were compared by the *χ*2 test or Fisher's exact test. Statistical significance was evaluated at *P* ≤ 0.05, and all analyses were performed using SPSS (version 25.0: SPSS, Inc., Chicago, United States) or R (version 3.5.1: R Foundation, Vienna, Austria).

## 3. Results

### 3.1. Patients and Treatments

**Figure 1:**
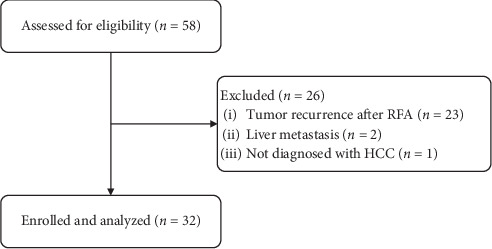
Patient selection.

Between May 1^st^, 2011, and August 1^st^, 2018, a total of 58 patients received RFA followed by liver SBRT at the SYSUCC. Among them, 23 were confirmed to have tumor recurrence rather than an iIR to RFA and were therefore excluded. In the remaining 35 patients, 2 had liver metastases, and 1 had intrahepatic cholangiocarcinoma. Finally, 32 patients were included in the study in accordance with the inclusion criteria (Figure 1). The longest follow-up was 5 years. As of April 1^st^, 2019, seven patients had died due to tumor progression, while the rest were treated as censored. The median follow-up time was 24.0 months (1.7 months to 60.0 months).

Hepatitis B virus (HBV) infection was present in 22 (68.8%) patients in this study, practically all of whom had received anti-HBV therapy. Treatments for hepatitis C virus (HCV) were not conducted in the 2 HCV-infected patients before SBRT and were executed after completion of the SBRT schedules. Although nearly half of the patients (46.9%) developed cirrhosis, their liver function remained compensated, and none of them scored 7 or worse on the CP scale. The median tumor diameter was 28 mm. Twenty-three lesions were classified as perivessel HCC, 8 were classified as subcapsular HCC, and 3 were both perivessel and subcapsular HCC. The baseline characteristics of the enrolled patients and the target lesions are listed in Table 1.

### 3.2. Tumor Control and Overall Survival

**Figure 2:**
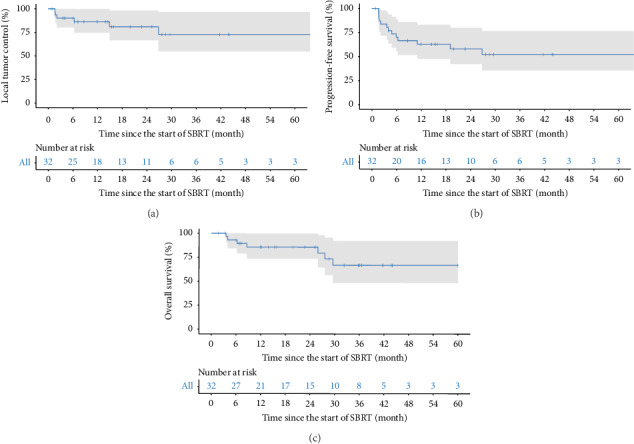
(a) Local tumor control of SBRT. (b) Progression-free survival of SBRT. (c) Overall survival of SBRT.

An objective response was observed in 8 (25.0%) of 32 lesions one month after completing SBRT treatment. At this time, the best overall responses were observed in 7 (21.7%) patients with complete response (CR) and 1 (3.1%) patient with partial response (PR). Twenty-one (65.9%) lesions were assessed as stable disease (SD), and 3 (9.3%) lesions were assessed as progressive disease (PD). During long-term follow-up, LTP was observed in 6 patients, 13 patients reached a sustained CR, and 3 patients reached a sustained PR; the long-term objective response rate (ORR) was 50.0%. The one- and two-year LTC rates were 86.6% (95% confidence interval (95% CI), 74.3% to 98.9%) and 74.7% (95% CI, 55.9% to 93.5%), respectively (Figure 2(a)). The median time to LTP was not reached. Univariate and multivariate analyses revealed that the number of prior treatments was associated with LTC: those who had received more than three prior treatments were more likely to suffer LTP (HR = 11.7, *P*=0.026, Table 2). However, the dose delivered to the PTV and the tumor characteristics, such as tumor location and diameter, did not contribute to LTC.

**Figure 3:**
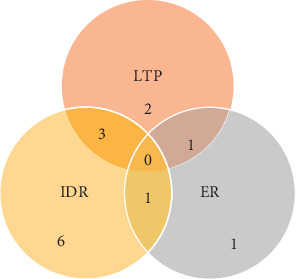
Composition of patients with tumor progression. LTP, local tumor progression; IDR, intrahepatic distance recurrence; ER, extrahepatic recurrence.

The composition of 13 patients with tumor progression is represented in Figure 3, including LTP in 6 patients, IDR in 10 patients, and ER in 3 patients. The median time to progression was not reached either. The one- and two-year PFS rate were 69.9% (95% CI, 53.4% to 86.4%) and 52.7% (95% CI, 33.1% to 72.3%) (Figure 2(b)), respectively. Multivariate analysis identified higher CP score as the only independent risk factor for PFS (HR = 5.17, *P*=0.012, Table 2). Details regarding the lesions showing tumor progression and further treatment modalities are listed in Table 3.

Seven patients died due to tumor progression in the follow-up period. The survival time after SBRT in these seven patients ranged from 4.0 to 29.6 months. The mean survival time of the enrolled patients was 82.9 months (95% CI 78.4–87.4 months), while the median survival time was not reached. The cumulative 1- and 3-year OS were 85.6% (95% CI, 72.5% to 98.7%) and 67.1% (95% CI, 45.7% to 88.5%) (Figure 2(c)). We included LTC as a dependent parameter and found no association between LC and better OS (HR = 0.85, *P*=0.88, Table 2). Age, BMI, liver function, number of prior liver-directed treatments, tumor characteristics, and radiation dosage were not associated with OS.

The results of the univariate and multivariate analyses are shown in Table 2.

### 3.3. Tolerance and Toxicity

All patients completed their SBRT schedule, and no dose reduction occurred. According to CTCAE 5.0, almost all patients (28 in 32) experienced various sorts and degrees of side effects; among them, only two patients suffered grade 3 adverse events and no patients experienced grade 4 or 5 events. Adverse events are shown in Table 4. The most common adverse event was decreased platelet count (40.6%), followed by increased bilirubin (37.5%) and increased aspartate aminotransferase (34.4%). No instances of RILD were observed. Only one patient suffered from liver function deterioration, which appeared as a progression of the CP score from 5 to 6 within one month after SBRT. We divided patients into two groups according to the median dose added to the PTV, and Fisher's exact test revealed that there was no correlation between the radiation dosage or liver function and the incidence of adverse events (*P*=0.63 or 0.56, respectively). Patients who received a higher radiation dosage or had worse liver function did not have a higher risk of enduring adverse events. Furthermore, whether toxicity occurred was not associated with better LTC, PFS, or OS.

## 4. Discussion

Thermal ablation, especially RFA, is recommended in HCC guidelines as one of the curative options for BCLC 0 or A-stage tumors, and the reported iCR rate of RFA ranges from 83.1% to 94.8% [3, 6–8]. Margarita Sala et al. revealed that achievement of an iCR is a major predictor of patient survival after RFA because it is associated with a significant outcome improvement [4]. Tumor size and stage are the most important predictors of an iCR, as the iCR rate reaches approximately 96% in tumors smaller than 2 cm and decreases to almost 50% in case of multinodular or large HCC lesions [4, 22, 23]. In addition, when blood flow is present in the vicinity of the ablation zone, the temperature declines significantly; thus, an iCR is less likely to be achieved. [5]. In most cases, patients receive repeated RFA after the failure of previous RFA procedure [8, 24], under which circumstance the limitations mentioned above are not truly resolved. In this observational study, we demonstrate that the strategy of giving individualized SBRT in 6 fractions to patients with a confirmed iIR to previous RFA provided promising tumor control and OS with tolerable adverse events. Six of the 32 patients suffered local progression, and the 1- and 2-year LTC rates were 86.6% and 74.7%, respectively. Although the LTC was encouraging, SBRT showed less efficiency in overall tumor control; IDR and ER occurred in more than one-third of the enrolled patients, which might have contributed to the unsatisfactory OS. In contrast, studies in which patients enjoyed 2-year post-SBRT LTC and OS rate of 74% to 95% and 30% to 46%, respectively, enrolled large numbers of treat-naïve patients, and patients with residual tumor after RFA composed a small part of their samples [11, 14, 17, 20]. Taking the relatively large heterogeneity of those study populations and the underlying distinct response to SBRT into account, we deemed that our study is more representative of patients with an iIR after RFA as well as the ability of SBRT to treat such residual lesions than the investigations mentioned above.

It has been reported that there exists a radiation dose-response relationship in HCC: a higher, more intense dose of SBRT likely contributes to a higher rate of LTC [20, 25]. However, a higher dose delivered to the PTV was not associated with better LTC in the univariate or multivariate analyses in our study. These findings seem contradictory because if a dose-response relationship did exist, then a higher radiation dosage should result in better LTC. A rational explanation could be that, in this investigation, we enrolled a heterogeneous group of patients in terms of the number of prior liver-directed treatments (median, 3; range, from 1 to 14), which indicated that the biological and behavioral characteristics of each tumor nodule differed greatly. Hence, sensitivity to radiation was apparently unique in every malignant lesion, and the benefit of a higher dose in terms of tumor control was concealed. In comparison to previous study, Tse et al. planned an irradiation of 24 Gy to 54 Gy (median 36 Gy) in six segmentations, the 1-year control rate was 65%, along with five patients (12%) having grade 3 liver enzyme increases [13]. A trial conducted by Cárdenes et al. reported that when starting at 36 Gy in 3 fractions, the 1-year control rate reached 100%, with two patients suffering grade 4 toxicity and three suffering classic RILD [26]. Kang et al. demonstrated that as the radiation dose was increased to 60 Gy in 3 fractions, the 2-year local control rate improved markedly to 94.6%, whereas 2 patients (4.3%) experienced grade 4 gastric ulcer perforation [27]. In our study, we prescribed a mean dosage of 42 Gy (30 Gy to 54 Gy) and a 6-fraction scheme. The 2-year LTC rate was 74.7%, without grade 4 or 5 adverse events or RILD. Combining our research findings with those reported by others, it seems that a lower radiation dose is more tolerable but less efficient for LTC, whereas a higher dose provides better tumor control but with a corresponding higher toxicity. Thus, we recommend 42 Gy in 6 fractions as a reference schedule for patients with residual diseases after RFA.

Multivariate analysis identified fewer prior treatments to be associated with better LTC. A similar conclusion was drawn in a clinical trial at the University of Michigan Medical Center, where fewer prior liver-directed therapies was related to longer survival [17]. Patients with an iIR after previous RFA received SBRT in our study, but two-thirds of them had received liver-directed therapies before RFA; in other words, two-thirds of the included patients had sustained tumor relapse. HCC in those patients tended to be more aggressive and less responsive to treatments. Accordingly, local tumor recurrence was more likely to occur in those patients. An interesting finding is that LTC had little impact on OS when LTC was included as a variable in the OS analysis (HR = 0.85, *P*=0.88). Apart from the diverse stages at which the patients were treated, progression outside the PTV may partly explain this consequence, as even if a single HCC nodule had been permanently eliminated, others would almost inevitably arise [8, 28]. Thus, combining SBRT with other regional or systemic therapies appears to be rational. For the patient's sake, no matter which local therapy has been adapted, SBRT, RFA, or resection, the regional tumor control rates have been proven similar [2, 7, 11–13, 17, 28–30]. Therefore, more effective systemic therapies and early recurrence-detection methods are needed.

The CP score was the only significant factor associated with tumor progression in our multivariate analysis. In our study, enrolled patients were all CP class A, yet those who were rated as CP score 6 were still more likely to suffer tumor progression compared to CP score 5. A similar finding was reported by Kwon et al., whose study demonstrated the CP score as a significant factor affecting not progression but survival [31]. While health status, tumor burden, and liver function greatly affect the prognosis of HCC patients [2], the CP score was once considered the strongest prognostic indicator for HCC [32, 33]. Our result is consistent with the consensus that tumor control is superior in patients with well-preserved liver function (CP score 5 patients) to those with mild function impairment (CP score 6 patients). Liver function was correlated with tumor control after SBRT in CP class A patients, but what about CP class B or C patients? Most prior studies have recruited CP class A patients, but Culleton et al. carried out a trial that recruited HCC patients with CP class B or C liver function. In their trial, the median survival of CP score 7 patients was 9.9 months versus 2.8 months for those CP score>7 (*P*=0.011), but the difference in time to progression did not reach statistical significance. Moreover, liver toxicity occurred more frequently in patients with CP class B or C liver function. In a phase I study conducted by Cardenes, all CP class A patients received 48 Gy of SBRT without dose-limiting toxicity, while 3 of 11 CP class B patients suffered DLT under 42 Gy of radiation [26]. Another retrospective study reported that 2 of 4 CP class B patients developed RILD following 35 Gy and 40 Gy of SBRT [34]. Those studies revealed that CP class B or C patients benefit less but risk more from SBRT; thus, we suggest that SBRT should be considered with caution in patients with high CP scores, especially CP class B or C patients.

The incidence of grade 3 adverse events was relatively low in our series, and DLT and RILD were not observed. The two patients who had grade 3 anemia and a platelet decrease had baseline hemoglobin and platelet levels close to the lower limits. The adverse effect of SBRT to some degree led to hemoglobin and platelet fall-off; however, the patients' health status contributed to these consequences as well. The two patients' blood system disorders had recovered in the subsequent follow-up. Liver enzyme elevation and bilirubin increase were the most common forms of hepatobiliary toxicity. Liver failure after SBRT was not detected, and all patients were assessed as CP class A after treatment termination. One patient who received 48 Gy of irradiation sustained a CP score deterioration from 5 to 6 at the first follow-up. An increase in CP score has often been observed in SBRT studies [11, 13, 14, 17, 18, 27, 35], but the exact reasons for this change are not clear. Hepatocyte damage after radiation and tumor progression may contribute to liver function deterioration, which is further reflected by increased total bilirubin, hypoalbuminemia, or CP score progression.

This study has several limitations. As a retrospective observational study, selection bias was inevitable, and little was done to reduce selection bias; thus, our conclusion needs to be further verified by prospective trials. Because of the relatively small sample size, our heterogeneous patient group was not representative enough to achieve a strong statistical power in detecting the effects of the variables. Moreover, some patients were lost to follow-up, and consequently, the long-term influence of SBRT was not fully evaluated, and the survival benefits were not truly presented.

In conclusion, in patients confirmed to have an iIR to prior RFA and compensated liver function, six-fraction SBRT of 42 Gy can provide favorable local tumor control and overall survival along with acceptable toxicity. Our study provides a rationale for clinical trials of SBRT aimed at residual tumor tissue after RFA.

## Figures and Tables

**Table 1:** Baseline characteristics.

Characteristic	No.	%
Total	32	
Sex		
Male	31	96.9
Female	1	3.1
Age (year)		
Median (range)	59.5 (29–80)	
Liver disease		
HBV	22	68.8
HCV	2	6.2
No virus infection	8	25.0
Anti-HBV therapy		
Yes	20	90.9
No	2	9.1
Cirrhosis		
Yes	15	46.9
No	17	53.1
Child-Pugh score		
5	26	81.3
6	6	18.7
Prior treatments		
Median (range)	3 (1–14)	
RFA only	11	34.4
RFA and surgery	1	3.1
RFA and TACE	15	47.0
RFA, surgery, and TACE	5	15.5
AFP (ng/ml)		
Median (range)	49.9 (1.1–6245.0)	
≥200	12	37.5
<200	20	62.5
Tumor diameter (mm)		
Median (range)	28 (14–69)	
Tumor location^†^		
Perivessels	23	
Subcapsular	8	
Vessel invasion		
Yes	3	9.3
No	29	90.7
Irradiation dose (Gy)		
Median (range)	42 (30–54)	

BMI, body mass index; HCC, hepatocellular carcinoma; IHC, intrahepatic cholangiocarcinoma; RFA, radiofrequency ablation; TACE, transarterial chemoembolization; AFP, alpha-fetoprotein. † Some lesions can be both perivessel and subcapsular HCCs or neither perivessel nor subcapsular.

**Table 2:** Univariate and multivariate analysis of local tumor control, progression-free survival, and overall survival.

Variable	Local tumor control				Progression-free survival				Overall survival			
	Univariate		Multivariate^†^		Univariate		Multivariate^†^		Univariate		Multivariate^†^	
	HR (95% CI)	*P*	HR	*P*	HR	*P*	HR	*P*	HR	*P*	HR	*P*
Age (years)^‡^	0.61 (0.11–3.34)	0.56	—	—	1.19 (0.40–3.54)	0.76	—	—	3.45 (0.66–18.02)	0.14	—	—
BMI (kg/m^2^)^‡^	1.08 (0.22–5.39)	0.92	—	—	0.99 (0.33–2.94)	0.98	—	—	0.33 (0.06–1.75)	0.19	—	—
Cirrhosis	0.55 (0.10–3.00)	0.49	NA	NA	0.97 (0.32–2.88)	0.95	NA	NA	0.86 (0.19–3.88)	0.85	—	—
Antivirus	1.22 (0.22–6.70)	0.82	—	—	1.41 (0.43–4.58)	0.57	—	—	0.81 (0.21–6.32)	0.78	—	—
CP score	4.54 (0.74–28.11)	0.10	NA	NA	5.17 (1.43–18.64)	0.012	5.17 (1.43–18.64)	0.012	1.43 (0.17–12.05)	0.74	—	—
No. of prior^‡^	12.86 (1.46–113.0)	0.02	11.69 (1.35, 101.4)	0.026	2.27 (0.76–6.83)	0.14	NA	NA	1.73 (0.39–7.81)	0.47	—	—
Perivessel	0.29 (0.06–1.46)	0.13	NA	NA	0.63 (0.19–2.07)	0.45	—	—	0.43 (0.10–1.94)	0.27	—	—
Subcapsular	1.71 (0.31–9.40)	0.54			0.98 (0.27–3.56)	0.97	—	—	1.71 (0.33–8.83)	0.52	—	—
Diameter (mm)	0.90 (0.16–5.00)	0.91	NA	NA	1.34 (0.45–3.99)	0.60	NA	NA	3.05 (0.59–15.83)	0.19	—	—
Dose to PTV^‡^	1.34 (0.27–6.69)	0.72	—	—	1.11 (0.37–3.29)	0.86	—	—	0.51 (0.10–2.64)	0.42	—	—
AFP (ng/ml)^§^	1.74 (0.34–8.85)	0.51	NA	NA	1.59 (0.51–4.96)	0.43	NA	NA	0.83 (0.16–4.46)	0.83	—	—
AD	NA	0.46	—	—	3.04 (0.39–23.48)	0.29	—	—	1.95 (0.23, 16.49)	0.54	—	—
LTC	—	—	—	—	—	—	—	—	0.85 (0.10–7.10)	0.88	—	—

BMI, body mass index; CP score, child-Pugh score; no. of prior, number of prior treatments; PTV, patient-specific planning target volume; AFP, alpha-fetoprotein; AD, adverse events; LTC, local tumor control; NA, not applicable. † Variables with *P* values less than 0.1 in univariate analysis and those that may have an impact on tumor progression or survival based on clinical experience were included in the multivariate analysis using the forward LR method. ‡ For the variables age, BMI, number of prior treatments, and dose to the PTV, patients were dichotomized by the median values to transform quantitative data into two-category data. § Patients were divided into the low-AFP group (serum AFP < 200 ng/ml before SBRT) and the high-AFP group (serum AFP ≥ 200 ng/ml before SBRT).

**Table 3:** Details of patients and lesions with tumor progression.

ID	Lesion size (mm)	Dose (Gy)	Progression type	PFS time (months)	Further treatment	Follow-up time (month)	Current status
1	15	36	LTP	1.6	Ablation	25.9	DOD
2	38	39	IDR	1.6	Best Care	6.0	Lost
3	68	39	IDR	1.7	TACE	6.3	Lost
4	48	42	IDR	5.9	TACE	7.2	Lost
5	29	48	IDR	11.0	Ablation	35.8	AWD
6	25	42	IDR	19.1	Ablation	25.2	AWD
7	20	54	ER	3.6	Best Care	3.6	DOD
8	47	48	LTP + IDR	2.1	Best Care	3.4	Lost
9	69	45	LTP + IDR	6.3	TACE	12.2	Lost
10^†^	24	39	LTP + IDR	14.9 + 4.0^†^	TACE	36.6	AWD
11	28	45	LTP + IDR	26.2	LR	36.0	AWD
12	19	42	LTP + ER	1.7	CIK	1.7	Lost
13	41	45	IDR + ER	4.8	Best Care	13.9	Lost

Abbreviations: LTP, local tumor progression; IDR, intrahepatic distance recurrence; ER, extrahepatic recurrence; TACE, transarterial chemoembolization; LR, liver resection; DOD, dead as a result of disease; AWD, alive with disease; Lost, lost to follow-up. † This patient was confirmed to have IDR 4.0 months after SBRT and received TACE as a salvage therapy. LTP was confirmed 14.9 months after SBRT, and TACE was performed on this patient again.

**Table 4:** Toxicity of SBRT.

Category	Toxicity grade		
	Grade 1 No.	Grade 2 No.	Grade 3 No.
Nausea	1	0	0
Abdominal pain	1	0	0
Rash	2	0	0
Anemia	7	0	1
WBC decreased	5	2	0
PLT decreased	8	4	1
Hypoalbuminemia	8	0	0
Bilirubin increased	10	2	0
ALT increased	6	0	0
AST increased	10	1	0
INR increased	0	0	0

Abbreviations: WBC, white blood counts; PLT, platelet; ALT, alanine aminotransferase; AST, aspartate aminotransferase; INR, international normalized ratio.

## Data Availability

The data used to support the findings of this study are available from the corresponding author upon request.
